# Molybdenum Disulfide Nanosheets Decorated with Platinum Nanoparticle as a High Active Electrocatalyst in Hydrogen Evolution Reaction

**DOI:** 10.1186/s11671-021-03644-6

**Published:** 2022-01-10

**Authors:** Mina Razavi, M. Sookhakian, Boon Tong Goh, Hadariah Bahron, Eyas Mahmoud, Y. Alias

**Affiliations:** 1 Department of Chemistry, Faculty of Science, University Malaya Centre for Ionic Liquids, University of Malaya, 50603 Kuala Lumpur, Malaysia; 2 Department of Chemistry, Faculty of Science, University of Malaya, 50603 Kuala Lumpur, Malaysia; 3 Department of Physics, Faculty of Science, Low Dimensional Materials Research Centre, University of Malaya, 50603 Kuala Lumpur, Malaysia; 4 Faculty of Applied Sciences, Universiti Teknologi MARA, 40450 Shah Alam, Selangor Malaysia; 5 Department of Chemical and Petroleum Engineering, United Arab Emirates University, 15551 Al Ain, United Arab Emirates

**Keywords:** Platinum nanoparticles, Molybdenum disulfide, Electrocatalyst, Hydrogen evolution reaction, Acidic medium

## Abstract

Electrochemical hydrogen evolution reaction (HER) refers to the process of generating hydrogen by splitting water molecules with applied external voltage on the active catalysts. HER reaction in the acidic medium can be studied by different mechanisms such as Volmer reaction (adsorption), Heyrovsky reaction (electrochemical desorption) or Tafel reaction (recombination). In this paper, facile hydrothermal methods are utilized to synthesis a high-performance metal-inorganic composite electrocatalyst, consisting of platinum nanoparticles (Pt) and molybdenum disulfide nanosheets (MoS_2_) with different platinum loading. The as-synthesized composite is further used as an electrocatalyst for HER. The as-synthesized Pt/Mo-90-modified glassy carbon electrode shows the best electrocatalytic performance than pure MoS_2_ nanosheets. It exhibits Pt-like performance with the lowest Tafel slope of 41 mV dec^−1^ and superior electrocatalytic stability in an acidic medium. According to this, the HER mechanism is related to the Volmer-Heyrovsky mechanism, where hydrogen adsorption and desorption occur in the two-step process. According to electrochemical impedance spectroscopy analysis, the presence of Pt nanoparticles enhanced the HER performance of the MoS_2_ nanosheets because of the increased number of charge carriers transport.

## Introduction

Various environmental issues are the results of global activities and the consumption of fossil fuels. Therefore, there have been challenges to figure out sustainable, clean, and eco-friendly fuels to overcome this problem. An inspiring alternative energy carrier to replace fossil fuels is hydrogen (H_2_) which is clean and free from CO_2_ emission. Therefore, it is of great interest to produce H_2_ from renewable resources such as water [1–4]. Hydrogen could be obtained from the splitting of water, an energy-intensive process that requires 237 kJ/mol. Electrolytic water splitting is a process where an electrical current dissociates water into oxygen and hydrogen. This process entails substantial effort in discovering breakthrough electrolytes and electrodes that are low cost, efficient, long-lasting, and stable. Hydrogen evolution reaction (HER) is an excellent route to produce high purity (≈ 100%) hydrogen from water electrolysis [5, 6]. Therefore, an ideal catalyst must have high stability and be present to start proton reduction with minimum over potential and large cathodic current densities, which leads to enhancing the HER efficiency [7–10].

So far, the most effective HER electrocatalysts reported are Platinum (Pt) and its alloy because of rapid reduction kinetics, low overpotential, and small onset potential for high-efficiency energy conversion [11, 12] However, it is not adequate to use a large amount of Pt due to its low availability and high price as an electrocatalyst [13]. On the other hand, it can significantly enhance Pt electrocatalyst activity by reducing the size and increasing the surface area, allowing more atoms at the exterior and subsurface site to be involved in the catalytic process [14]. Therefore, control of Pt size and avoiding its agglomeration are favourable strategies to enance its electrocatalytic activity [15]. Consequently, it is very effective to deposit Pt nanoparticles with high homogeneity on the supporting materials such as conductive polymers, carbon, metal oxide, and sulfide[16] The supporting materials typically provide accessibility and stabilization platforms for nanoparticle growth and bring about modified reactivity and additional adsorption and active sites [17] Therefore, the decoration of Pt nanostructures such as nanostructure on supporting materials not only enhances the physiochemical properties of supporting materials but also prevents agglomeration of Pt, which influences the activity of composite towards HER [18–21]. Newly, earth plentiful source, high efficiency, and affordable transition metal dichalcogenides (TMDs) are considered good alternatives for HER. Synthesizing TMD nanomaterials as HER electrocatalysts requires considerable effort due to their noteworthy characteristics such as excellent electrochemical, optical, mechanical properties. They have low overpotential, small Tafel slope, and high air stability which exhibit high HER performance. Molybdenum disulfide (MoS_2_) and tungsten disulfide (WS_2_) are two materials among the TMDs group, which have electrical properties that can be altered from metallic to semiconducting by varying their crystal structure and the number of layers. MoS_2_ is one of the well-known and most widely investigated semiconductors which is an eco-friendly, non-toxic, and abundant semiconductor with an atomic-thickness layer structure similar to graphene [22–25]. HER performance of bulk MoS_2_ was investigated by J.C.Bennett et al. in 1977 and found that its activity is low, as evidenced by a large Tafel slope of 692 mV/dec and a high onset potential of about − 0.09 V versus HER. MoS_2_ nanoparticles were reported as HER active species by Hinnemann et al. in 2005. Afterward, MoS_2_ has taken back popularity, and a slew of new techniques have arisen to improve its HER electrocatalytic performance.[26]. According to the recent work, the HER activity of MoS_2_ is highly related to the exposed edges, while due to its semiconductive nature, it has poor conductivity [27]. To solve this problem, designing a novel composite based on MoS_2_ with the high exposed active sites and enhancing its conductivity is the key to elevating the properties of MoS_2_-based electrocatalysts for HER [2, 28] Moreover, MoS_2_ nanosheets possess good electrochemical stability and high surface area. They also function as effective support materials to efficiently decorate highly catalytic active species such as Pt nanoparticle to attain HER catalytic activity [29]

Herein, we successfully demonstrate a productive and straightforward hydrothermal method for synthesizing MoS_2_ nanosheets supported Pt nanoparticles (Pt/Mo). The as-synthesized Pt/Mo composites with different Pt loading were used as electrocatalyst for HER. Optimization of Pt loading on the surface of MoS_2_ nanosheet is an attempt to understand the effect of Pt loading on the electrocatalytic activity of MoS_2_ nanosheet in HER. This technique decreases Pt consumption in the composite because MoS_2_ nanosheets compensate Pt effects in the composite, which is a more affordable way in the hydrogen production economy.

## Experimental Methods

### Chemical Reagent

All chemicals that were used in this experiment, such as Chloroplatinic acid solution (H_2_PtCl_6_), Thioacetamide powder (C_2_H_5_NS), Ammonium heptamolybdate powder ((NH_4_)_6_Mo_7_O_24_) and Sulfuric acid solution (H_2_SO_4_) were purchased from Sigma Aldrich.

### Synthesis of MoS_2_ Nanosheets

Hydrothermal treatment was performed to synthesize MoS_2_ nanosheet, in which 188 mg of C_2_H_5_NS and 309 mg of (NH_4_)_6_Mo_7_O_24_ were taken and completely dissolved for an hour in 45 ml of distilled water. Later this colourless mixture was transferred into a stainless 45 ml Teflon-lined autoclave and kept at 180 °C for 18 h. After that, the black powder MoS_2_ was centrifuged and washed with distilled water three times. The final black powder product dried at 65 °C for a day.

### Synthesis of Pt/Mo Composites

In a typical process, 50 mg of MoS_2_ nanosheets were dispersed in 25 ml of deionized water under sonication for 30 min to get homogenous dispersion. Then, 60 μL of H_2_PtCl_6_ (8% in H_2_O) solution was dissolved in 20 ml of distilled water, and then the solution was added drop wisely to the MoS_2_ dispersion under sonication for an hour. The final mixture was heated at 70 ºC for 4 h. The as-synthesized Pt/Mo-x composites with *x* = 60, 90, and 120 μL of H_2_PtCl_6_ were referred to as Pt/Mo-60, Pt/Mo-90, and Pt/Mo-120 with 1.17 × 10^–2^ gr, 1.75 × 10^–2^ gr, and 2.34 × 10^–2^ gr Pt loading, respectively. The same procedure was done in the previous section for washing and drying Pt/Mo composite with different Pt loading.

### Physical and Chemical Characterization

The physical characterization of the material, such as crystal structure, size, and morphology, was studied by an X-ray diffractometer (XRD Smart Lab Guidance, Rigaku) and a High-resolution transmission electron microscope (HR-TEM Techno, FEI). Prior to drop-casting on the copper grid, the dilute and homogenous dispersion was prepared in DI water under sonication. The chemical analysis of the material, such as elemental composition and oxidation states, was conducted by X-ray photoelectron spectroscopy (XPS Model 1257, Perkin Elmer) and Raman (HR800 JY, Lab RAM HR) spectroscopy. The electrochemical measurements were carried out by galvanostat/potentiostat (Autolab PSTAGT50).

### Electrode Fabrication

Preparation of the electrode for electrochemical measurement was carried out by the drop casting method. Briefly, 5 mg of the different electrocatalysts was sonicated in 5 ml of distilling water for 30 min. Same methode was done to prepare solution of 20 wt% Pt/C for electrochemical mesearment. The working electrode utilized in this system is a glassy carbon electrode (GCE). Then, five μL of homogenous dispersion was drop casted on the surface of the clean GCE. The electrodes stayed at room temperature for complete dryness.

### Electrochemical Measurement

Electrochemical measurement was performed in the solution of 0.5 M H_2_SO_4_. Briefly, 15 ml of the 0.5 M H_2_SO_4_ solution was used as the electrolyte in the three-electrode cell. The electrocatalytic HER activities of pure MoS_2_, Pt/Mo-x composites with different Pt loading (*x* = 60, 90, 120), and commercial Pt (Pt/C) were studied in the 0.5 M H_2_SO_4_ solution by linear sweep voltammetry (LSV) and electrochemical impedance spectroscopy (EIS) techniques using a three-electrode cell. The as-synthesized samples-modified GCE was used as the working electrode. The reference and counter electrodes were chosen to be an Ag/AgCl electrode and a graphite rod, respectively. The polarization curves were obtained by sweeping the potential from 0.1 to − 0.6 V υs RHE at a potential sweep rate of 20 mV/s.

## Results and Discussion

### Structural and Morphological Analysis

**Fig.1:**
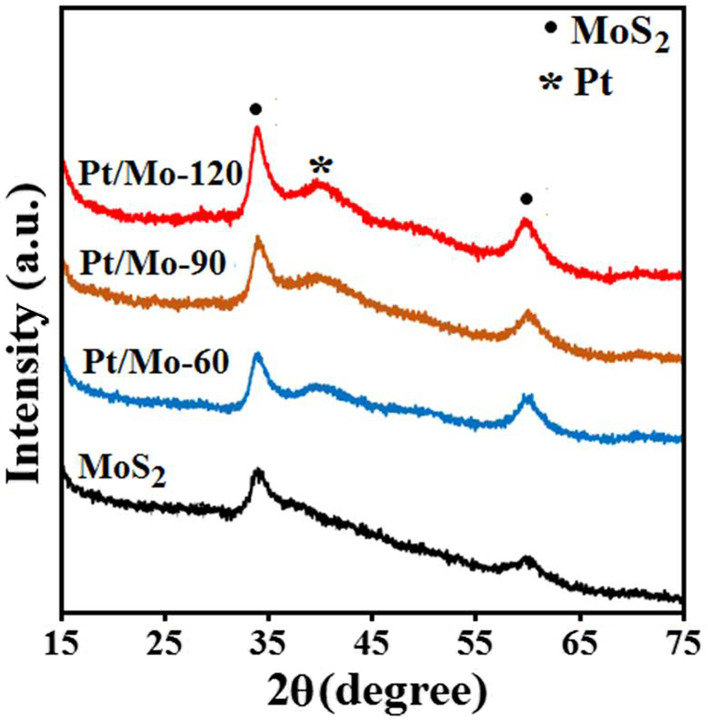
X-ray diffraction patterns of pure MoS_2_ nanosheets and Pt/Mo-*x* (*x* = 60, 90, 120) composites

X-ray diffractograms of the pure MoS_2_ nanosheets and Pt/Mo-x composites with three different Pt loading (*x* = 60, 90, 120) are shown in Fig. 1. The two peaks at 2*θ* values of 33.791° and 59.471° are indexed to the (100) and (106) planes, respectively (JCPDS Card No. 98–002-4000), of the hexagonal structure of MoS_2_ (2H phase) with lattice constants of *a* = 3.15 Å, *b* = 3.15 Å and *c* = 12.3 Å [22] However, compared with the pure MoS_2_ nanosheets, the X-ray diffractograms of the Pt/Mo-x composites with three different Pt loading exhibits an additional (111) peak at 2*θ* = 39.57° can be assigned to the (111) diffraction of cubic platinum (JCPDS card no. 00-004-0802) with the lattice constant *a* = *b* = 3.923 Å [29]. In addition, no other peaks were observed apart from the peaks of MoS_2_ and Pt, indicating a high phase purity of the Pt/Mo-x composites. Notably, the XRD results of Pt/Mo-x composites with three different Pt loading demonstrates that as Pt loading content in composite increases, the intensity of the peak at 2*θ* = 39.57° increases.

**Fig. 2:**
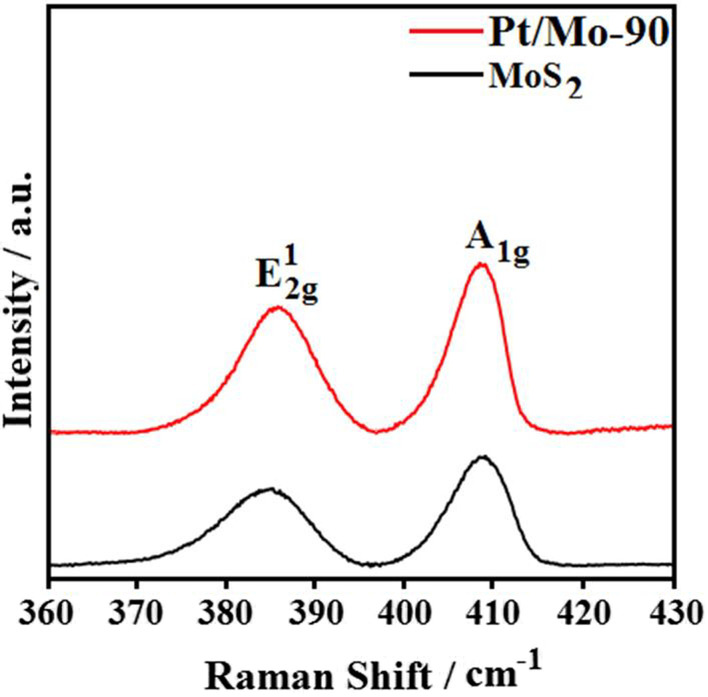
The Raman spectra of pure MoS_2_ nanosheets and Pt/Mo-90 composite

Raman spectroscopy technique is an efficient non-destructive chemical analysis to investigate the structural properties and the details of the thickness of MoS_2_ nanosheets. Figure 2 shows the Raman spectra of pure MoS_2_ nanosheets and Pt/Mo-90 composite, obtained over the range of 360–430 cm^−1^, where two longitudinal peaks of pure MoS_2_ at 384 cm^−1^ and 407 cm^−1^ are assigned to the $E_{2g}^{1}$ and *A*_1g_ modes. The former one could be attributed to the planar vibrations between S and Mo atom, while the latter one illustrates the vibration of sulfides in the out-of-plane direction [22]. Based on the peak position difference between $E_{2g}^{1}$ and *A*_1g_ modes which were calculated to be 23 cm^−1^, the MoS_2_ nanosheets were determined to be tri-layers [30] On the other hand, the Raman spectra of Pt/Mo-90 shows similar spectra compared to blank MoS_2_, but only an increase in peak intensity and slight red shift of both the $E_{2g}^{1}$ and *A*_1g_ peaks were observed in the Raman spectrum of Pt/Mo-90, implying a successful formation of the composite with the decoration of Pt nanoparticles on the surface of MoS_2_ nanosheets [31]. Noted, the slight red shift of phonon modes may be assigned to the heating of the composite during the Pt decoration [31].

**Fig. 3:**
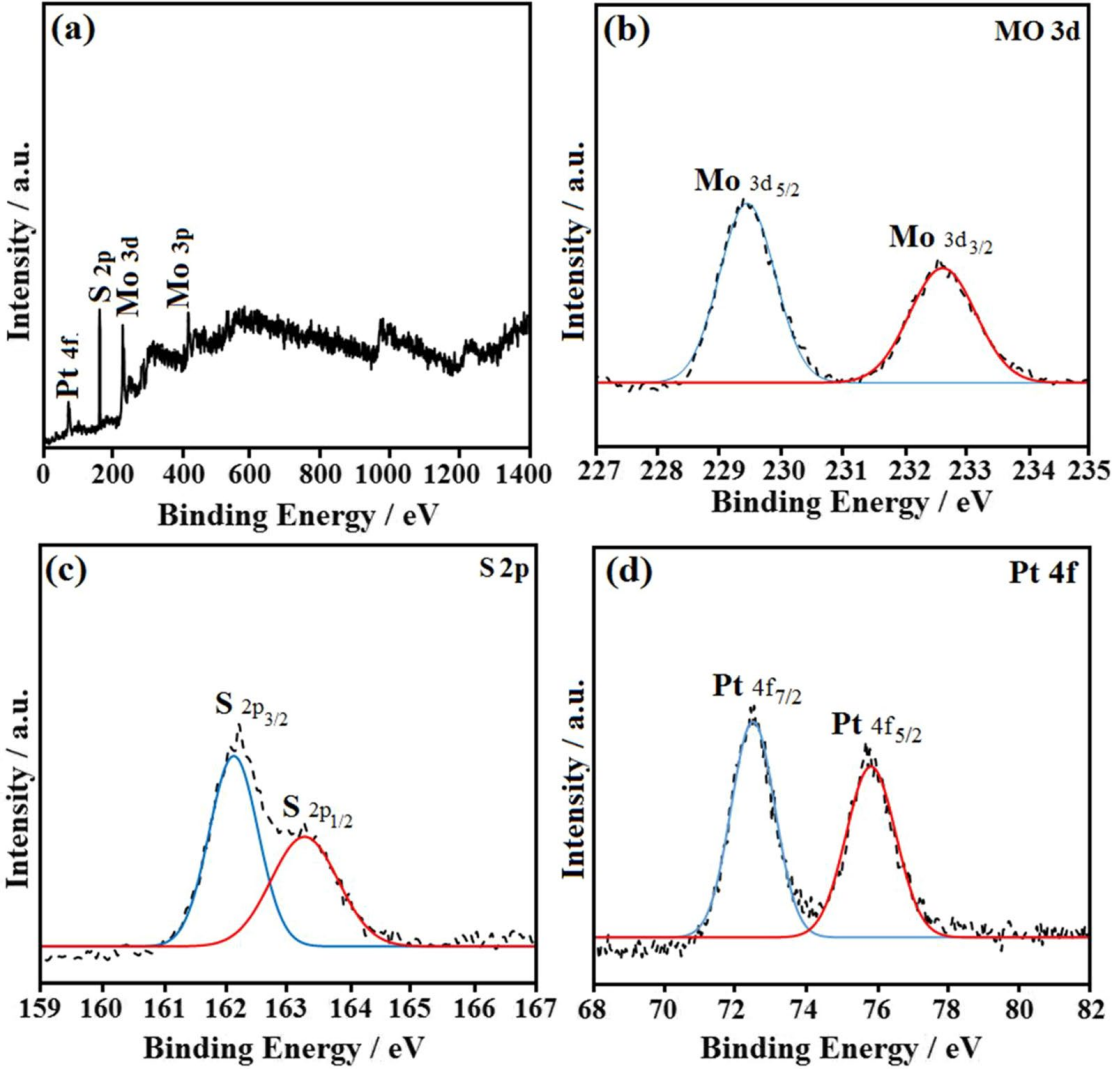
Wide scan XPS spectra of **a** Pt/Mo-90 composite; The high-resolution XPS spectra of **b** Mo 3*d*, **c** S 2*p*, and **d** Pt 4*f* of Pt/Mo-90 composite

The XPS analysis was performed to investigate the surface atom electronic structure of the Pt/Mo composite (Fig. 3). From the wide scan XPS spectrum of Pt/Mo-90 composite, the elements of sulfur (S), molybdenum (Mo), and Platinum (Pt) can be determined. As shown in Fig. 3a, four peaks obviously observed in the XPS survey spectrum of Pt/Mo-90 composite at 74.2 eV (Pt 4*f*), 162.4 eV (S 2*p*), 229.6 eV (Mo 3*d*), and 408.9 eV (Mo 3*p*). The atomic ratio value of S 2*p*: Mo 3*d* is estimated at 2.04, which reveals a successful hydrothermal process that leads to the formation of the MoS_2_ nanosheets [32]

The XPS spectrum corresponding to that of Mo 3*d* of the Pt/Mo-90 composite shows two strong peaks at 229.4 and 232.5 eV, which are referred to as the Mo 3d5/2 and Mo 3*d*3/2 doublet, respectively (Fig. 3b) [33]. Notably, the XPS spectra of Mo 3*d* clearly confirmed the existence of Mo (IV) in the MoS_2_ structure due to of presence of these two different strong peaks. The S 2*p* component of Pt/Mo-90 composite, which suggests S binding, exhibits two binding energies at 162.1 eV and 163.3 eV corresponding to the S 2*p*3/2 and S 2*p*1/2 in MoS_2_ structure (Fig. 3c) [33]. Besides, in the spectrum of Pt 4*f* of Pt/Mo-90 composite, two peaks were observed at 72.6 eV and 65.8 eV which are related to the Pt 4*f*7/2 and Pt 4*f* 5/2, respectively (Fig. 3d) [34].

**Fig. 4:**
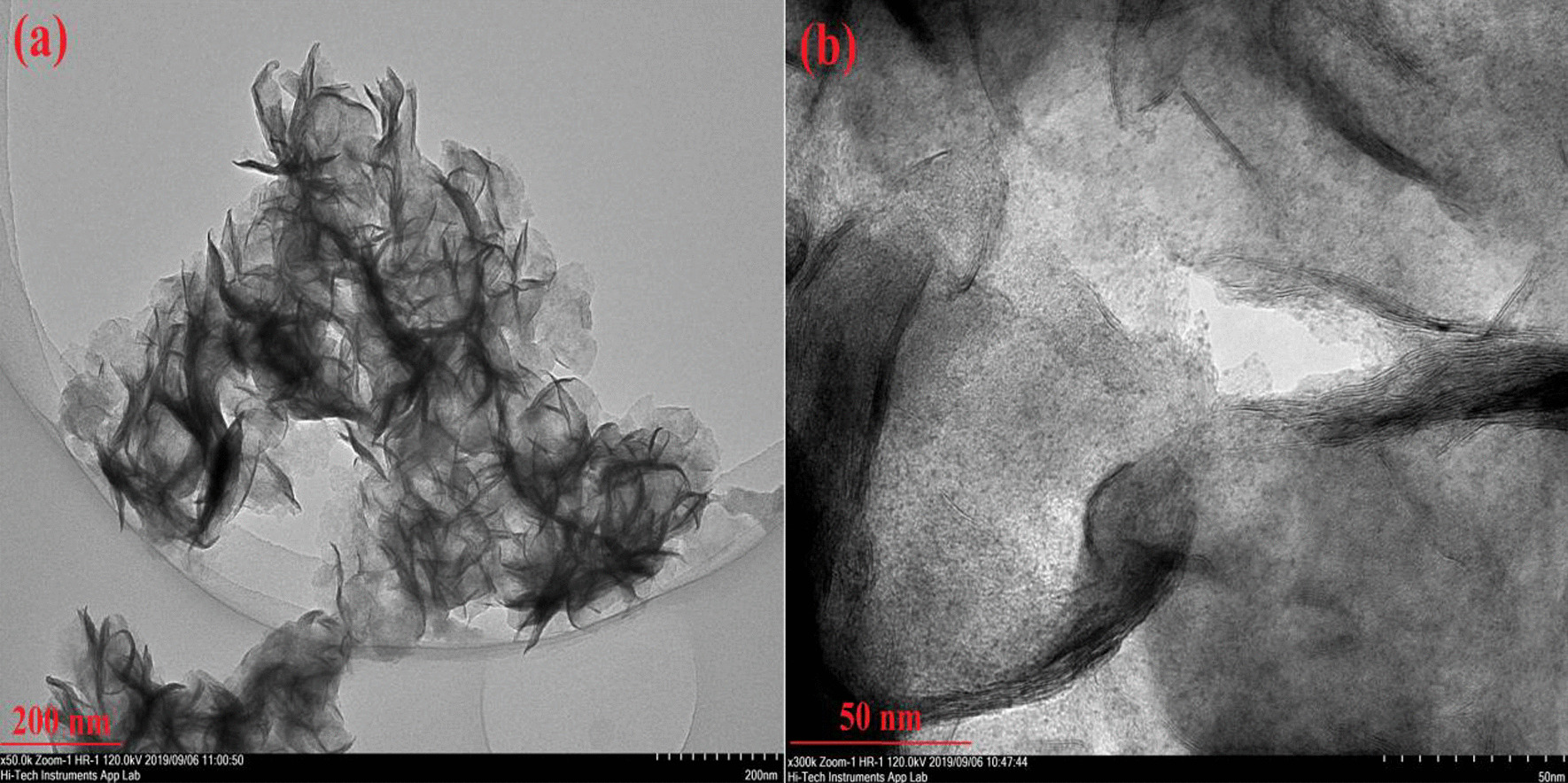
**a**, **b** TEM image of Pt/Mo-90 composite

**Fig. 5:**
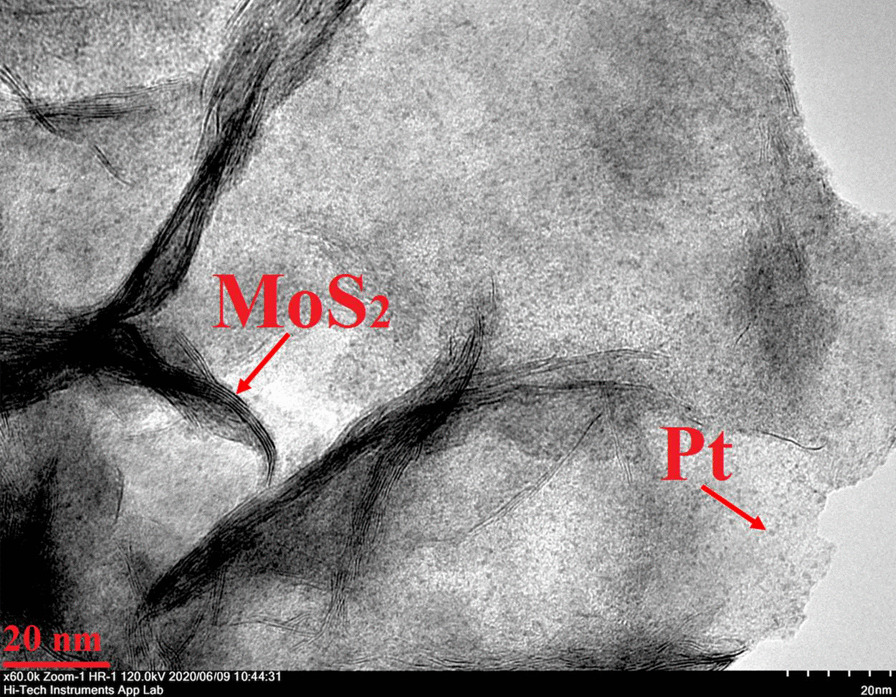
High magnification TEM image of Pt/Mo-90 composite

The morphology of the as-synthesized Pt/Mo-90 composite was carried out by TEM analysis (Fig. 4 and 5). Figure 4a represents the TEM image of the as-synthesized Pt/Mo-90. The average diameter of the as-synthesized Pt/Mo-90 was obtained 1 μm, and the surface structure of the nanosheet is wrinkle shape. According to Fig. 4b, in Pt/Mo-90 the Pt nanoparticles have grown uniformly on the surface of MoS_2_ nanosheet.

Figure 5 shows the high-resolution TEM image of Pt/Mo-90 composite in which Pt nanoparticles are successfully decorated on the surface of the MoS_2_ nanosheet having an average size of 2 nm. Notably, Fig. 5 confirmed that some of the Pt nanoparticles being also located between the crumpled MoS_2_ nanosheets.

### Catalytic Performance for HER

**Fig. 6:**
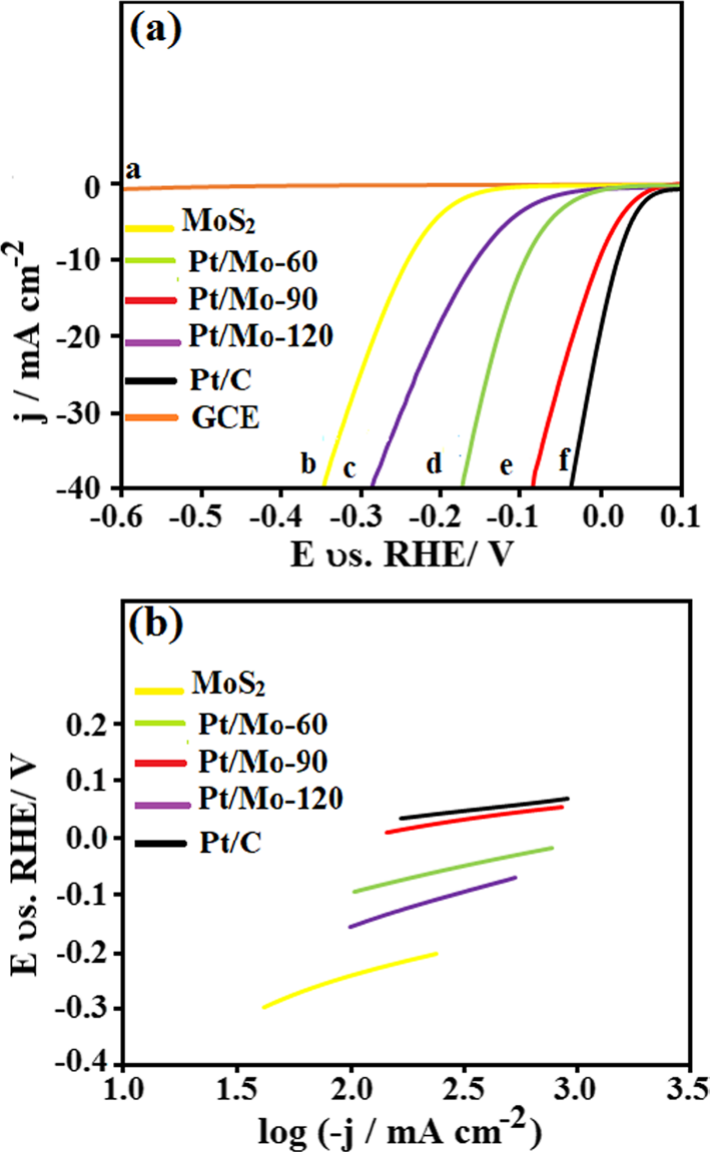
**a** LSV curves of **a** bare GCE, **b** Pure MoS_2_ nanosheets, **c** Pt/Mo-120, **d** Pt/Mo-60, **e** Pt/Mo-90 composite, and **f** Pt/C; **b** Tafel plot of different electrocatalyst

To obtain knowledge about the effect of Pt loading on HER electrocatalytic performance of MoS_2_ nanosheet, the linear sweep voltammetry (LSV) was performed in N_2_-saturated 0.5 M H_2_SO_4_ using a three-electrode electrochemical cell. Figure 6a shows the polarization curves of different electrocatalysts such as bare GCE, pure MoS_2_ nanosheets, Pt/Mo-*x* (*x* = 60, 90, 120), and 20 wt.% Pt/C-modified GCE at the scan rate of 20 mV s^−1^. There is not any HER activity observed from the bare GCE; hence the effect of GCE is neglected.

According to Fig. 6a, the presence of Pt nanoparticles causes in enhancing of the onset potential, half-wave potential, and overpotential of MoS_2_ nanosheets. However, with the increase of the Pt loading up to 90 μl, the LSV curve initially enhances for Pt/Mo-90, but further increase in Pt loading up to 120 μl leads to worsening of the LSV curve. Based on the LSV curve, at a current density of − 10 mA cm^−2^, the pure MoS_2_ nanosheets, Pt/Mo-120, and Pt/Mo-60 composites exhibit onset potential of − 0.15, − 0.03, and − 0.01 V, respectively, while Pt/Mo-90 and Pt/C show a remarkable onset potential of + 0.05 and + 0.07 V, respectively. Another prominent factor in HER activity is the half-wave potential (the potential where the current is half of the limiting current). According to Fig. 6a, the half-wave potential of Pt/Mo-x (*x* = 60, 90, 120) composite electrodes showed a more positive potential compared with pure MoS_2_ nanosheets because of the presence of Pt nanoparticles in composites. Moreover, the Pt/Mo-90-modified GCE at the current density of − 10 mA cm^−2^ showed a lower overpotential of − 0.01 V, comparing with the pure MoS_2_-modified GCE (− 0.24 V), Pt/Mo-120-modified GCE (− 0.16 V), and Pt/Mo-60-modified GCE (− 0.09 V), respectively. Notably, the overpotential value of Pt/Mo-90 (− 0.01 V) was very near to that of 20 wt.% Pt/C (+ 0.01 V), showing that the composite electrocatalyst had Pt-like electrocatalytic activity.

The Tafel plot is another important metric in HER. It is utilised to investigate the kinetics of the materials' HER electrocatalytic activity. Figure 6b exhibits the Tafel plots of the different electrocatalysts. As observed, among all electrocatalyst composites, Pt/Mo-90-modified GCE exhibited the smallest Tafel slope of 41 mV dec^−1^, which is very near to the commercial of 20 wt.% Pt/C (37 mV dec^−1^). Pt/Mo-60 (75 mV dec^−1^) and Pt/Mo-120 (112 mV dec^−1^) showed larger Tafel slop comparing to Pt/Mo-90 while lower than pure MoS_2_ nanosheet (126 mV dec^−1^). According to the earlier works, HER reaction in the acidic medium can be studied by three different mechanisms. Volmer reaction is the first one, in which the source of the proton is the hydronium ion (H_3_O^+^) for the primary discharge step [35]:

1 $$\begin{aligned} & H_{3} O^{ + } + e^{ - } \to H_{ads} + H_{2} O \\ & b = \frac{2.3RT}{{\alpha F}} \approx 120\;{\text{mV}} \\ \end{aligned}$$

Based on the above formula, *b* is the Tafel slope, *F* is the Faraday constant, *R* is the ideal gas constant, *α* is the symmetry factor ( ≈ 0.5), and T is the temperature. Heyrovsky reaction (Electrochemical desorption) or Tafel reaction (recombination) will occur in the following steps as shown by Eq. 2 and 3, respectively.

2 $$\begin{aligned} & H_{ads} + H_{3} O^{ + } + e^{ - } \to H_{2} + H_{2} O \\ & b = \frac{2.3RT}{{(1 + \alpha )F}} \approx 40\;{\text{mV}} \\ \end{aligned}$$

3 $$\begin{aligned} & H_{ads} + H_{ads} \to H_{2} \\ & b = \frac{2.3RT}{{2F}} \approx 30\;{\text{mV}} \\ \end{aligned}$$

According to the as-calculated Tafel slope of Pt/Mo-90 composite (41 mV dec^−1^), the HER mechanism is related to the Volmer-Heyrovsky mechanism, where hydrogen adsorption and desorption occur in the two-step process.

### Durability and Stability

**Fig. 7:**
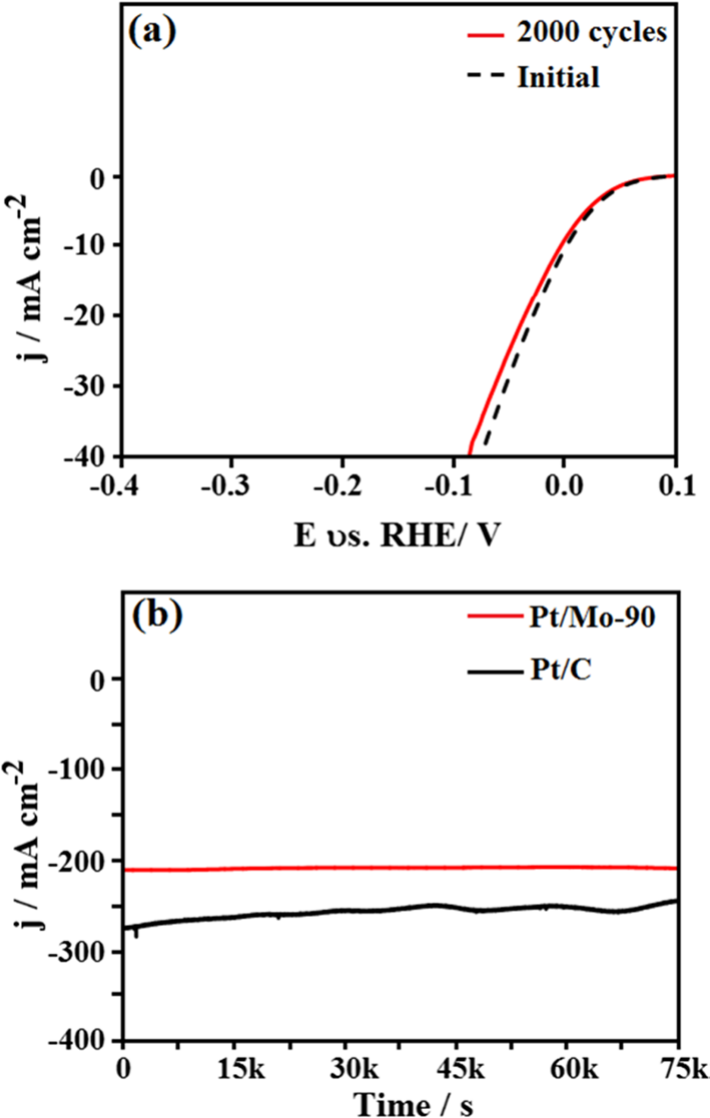
**a** LSV curves of Pt/Mo-90 composite prior to and posterior to 2000 CV cycles; **b** The chronoamperometric response of Pt/Mo-90 and Pt/C under − 0.25 V

Two key factors to investigate the HER electrocatalytic activity are long-term stability and durability. Therefore, to study the as-synthesized Pt/Mo-90 composite durability, continuous cyclic voltammogram (CV) scanning was performed in 0.5 M H_2_SO_4_ as an electrolyte under the scan rate of 50 mV s^−1^. As seen in Fig. 7a, the LSV curve of Pt/Mo-90-modified GCE even after 2000 cycles does not show any changes or drift, which indicates that the as-synthesized Pt/Mo-90 composite has high durability.

The stability of the electrodes is another critical parameter in HER application. For this reason, the chronoamperometric (current vs. time) response of the as-synthesized Pt/Mo-90 composite was done in N_2_-saturated 0.5 M H_2_SO_4_ electrolyte (Fig. 7b). As seen in Fig. 7b, the as-synthesized Pt/Mo-90 composite shows high stability during 20 h of the experiment at a constant potential of − 0.25 V, which is more stable than Pt/C. Table 1 demonstrates some of the HER activity parameters such as overpotential, Tafel slope, and stability of the Pt/Mo-90 and some of the recently reported composites. As seen, the Pt/Mo-90 composite is comparable to that of the other electrocatalysts.

**Table 1:** A Summary and a comparison of the current work with earlier studies in the literature

Modified electrode	Electrolyte	*V*_over_ (mV)	Tafel slop (mV dec^−1^)	Stability	References
Rh-MoS_2_	0.5 M H_2_SO_4_	− 47	24	20 h	[36]
MoS_x_@NiO	1 M KOH	− 406	43	13 h	[37]
Co-WS_2_	0.5 M H_2_SO_4_	− 134	76	–	[38]
FeP/C	0.5 M H_2_SO_4_	− 95	74	15 h	[39]
MoS_2_	0.5 M H_2_SO_4_	− 194	59	9 h	[40]
Pt/CoSe	1 M PBS	− 19	35	40 h	[41]
Ni-CoCHH/NF-S	1 M KOH	− 100	40	25 h	[42]
MoC@GS	0.5 M H_2_SO_4_	− 132	46	10 h	[43]
Pt/Mo-90	0.5 M H_2_SO_4_	− 10	41	20 h	This work

**Fig. 8:**
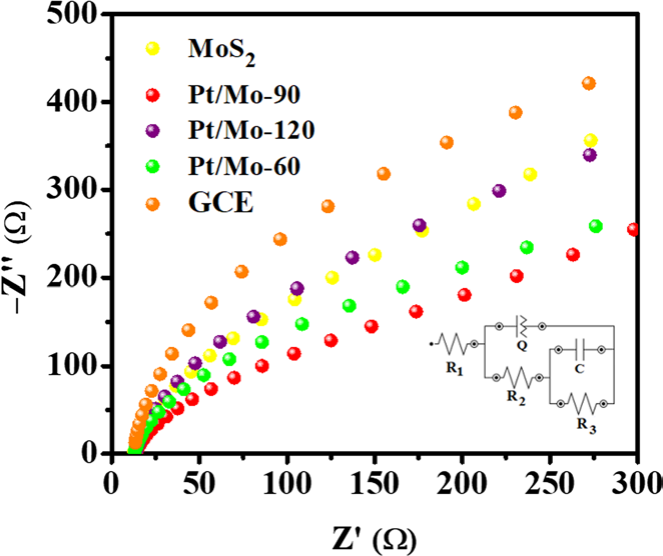
Nyquist plots of bare GCE, Pure MoS_2_ nanosheets, Pt/Mo-60, Pt/Mo-90, and Pt/Mo-120-modified GCE in 0.5 M H_2_SO_4_ solution; inset: the equivalent circuit

As seen in LSV curve and Tafel, Pt nanoparticles increased the HER activity of MoS_2_ nanosheets in the Pt/Mo-*x* (*x* = 60, 90, 120) composites This enhancement could be due to the synergistic interaction between the MoS_2_ nanosheets and Pt nanoparticles which are active the materials and results in increasing, the number of charge carriers transport. Therefore, to examine the kinetic interactions at the cathode, electrochemical impedance spectroscopy (EIS) was done in the frequency range of 0.1–10^5^ Hz at a five mV AC signal amplitude (the reaction between ion diffusion and electrode). Figure 8 shows the Nyquist plots of the bare GCE, pure MoS_2_, Pt/Mo-60, Pt/Mo-90, and Pt/Mo-120-modified GCE in 0.5 M H_2_SO_4_ electrolyte. As shown in the inset of Fig. 8, the EIS curves were simulated using the non-linear least square method according to the equivalent circuit. On the basis of the fitting circuit, different parameters were observed, such as *R*_*1*_ (solution resistance), *R*_*2*_ (charge transfer resistance), *Q* (constant phase element), and *C* (double-layer capacitance).

A semicircle at the low-frequency region is because of a charge transfer between the electrolyte and the electrode. As is seen in Fig. 8, a semicircle can be observed at a low-frequency region, revealing that Faradaic charge transfer is happening among the electrolyte and the cathode interface (*R*_*2*_). Getting a satisfactory correlative between the simulated complex circuit with the experimental data, Q and *R*_*3*_ components were introduced to the circuit.

As is shown in Table 2, pure MoS_2_ and the Pt/Mo-*x* (*x* = 60, 90, 120) composites exhibit almost the same solution resistances (*R*_*1*_). However, the *R*_*2*_ of the Pt/Mo-90 composite is smaller than the pure MoS_2_ (Table 2). This indicates that the electrocatalytic activity of the MoS_2_ nanosheets was increased by the presence of Pt caused by the increased number of charge carriers transport, resulting in a faster rate of charge transfer. However, the further increment of Pt loading beyond the optimum level of 90 μL caused a decrease of catalytic activity, as shown by the increased *R*_*2*_ value of the Pt/Mo-120-modified GCE electrode (Fig. 8 and Table 2). It was indicated that excessive loading of Pt could lead to agglomeration of Pt nanoparticles during synthesis, causing reduced surface area and catalytic active sites. This increase in *R*_2_ value brought about a significant decrease in HER catalytic performance.

**Table 2:** Electrochemical parameters achieved from the simulation of the EIS results

Electrode	*R*_1_	*R*_2_	*R*_3_	*C*	*Q*	*n*
	(Ω cm^2^)	(Ω cm^2^)	(Ω cm^2^)	(μF cm^2^)	*Y*_0_ (μ Ω^−1 ^s^n^cm^−2^)	
MoS_2_-GCE	35.6	996	4.5 × 10^3^	122	10.60	0.80
Pt/Mo-60-GCE	38.1	242	1.59 × 10^3^	83	4.08	0.85
Pt/Mo-90-GCE	34.8	147	1.22 × 10^3^	27	2.83	0.89
Pt/Mo-120-GCE	35.1	635	1.97 × 10^3^	31	2.63	0.77

To understand better the mechanism of electron transfer, we propose here a band alignment of the most efficient Pt/Mo-90 composite device.

**Fig. 9:**
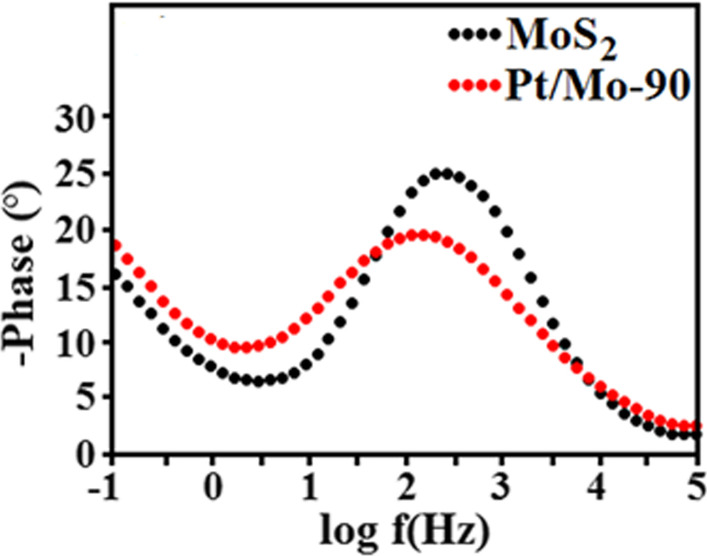
Bode EIS plots of the pure MoS_2_ and Pt/Mo-90 composite

Figure 9 shows the effect of Pt nanoparticles on the electron–hole recombination of the HER devices that could be obtained from the Bode EIS plot [44]. The electron lifetime ($\tau_{r}$) can be estimated using the frequency $f_{p}$ at the middle frequency peak (1–100 Hz) in the Bode phase plot from the following equation [45]

4 $$\tau_{r} = \frac{1}{{2\pi f_{p} }}$$

Based on the Bode plot (Fig. 9, it is obvious that the electron lifetime of Pt/Mo-90 composite is higher than pure MoS_2_ because of the lower $f_{p}$. Therefore, presence of Pt nanoparticles in composite has enhanced electron lifetime which leads to improve the HER activity of the device.

## Conclusion

In conclusion, a hydrothermal method is utilized to synthesis MoS_2_ nanosheets with an average diameter of 1 μm. At the later stage, Pt nanoparticles having a diameter of 2 nm on average and different Pt content were deposited onto MoS_2_ nanosheets to produce Pt/Mo-*x* (*x* = 60, 90, 120) composites as electrocatalysts for the HER. XRD micrographs, XPS, TEM, and Raman support the presence of Pt nanoparticle on the surface of MoS_2_. Among these different electrocatalysts, the Pt/Mo-90- modified GCE showed excellent electrocatalytic activity, stability, and durability for HER application. Regarding the overpotential, the Pt/Mo-90 composite showed Pt-like activity with an overpotential of only − 0.01 V μs. RHE to reach a current density of − 10 mA cm^−2^ in 0.5 M H_2_SO_4_. Moreover, in comparing with the Tafel slop of Pt/C (37 mV dec^−1^), the Pt/Mo-90 composite exhibited the smallest Tafel slope of 41 mV dec^−1^. This composite showed good long-term stability after 20 h as well. The EIS measurement confirmed that the electrodes' electrocatalytic activity depends on the amount of Pt loading; increasing the Pt loading up to 120 μl leads to a rise in charge transfer resistance of the composite electrode, which results in a decrease in the HER electrocatalytic activity.

## Data Availability

All the data and material are available in the manuscript.

## Abbreviations

Pt: Platinum
Pt/Mo: Platinum molybdenum disufide
MoS_2_: Molybdenum disulfide
H_2_: Hydrogen
HER: Hydrogen evolution reaction
TMDs: Transition Metal dichalcogenides
H_2_PtCl_6_: Chloroplatinic acid solution
C_2_H_5_NS: Thioacetamide powder
((NH_4_)_6_Mo_7_O_24_): Ammonium heptamolybdate powder
(H_2_SO_4_): Sulfuric acid solution
H_2_O: Water
Pt/Mo-*x* (*x* = 60, 90, 120): Platinum molybdenum disufide composites with different Pt loading
LSV: Linear sweep voltammetry
EIS: Electrochemical impedance spectroscopy
RHE: Reversible hydrogen electrode
Pt/C: Commercial Platinum 20 wt.%
GCE: Glassy carbon electrode
2H: Hexagonal
XRD: X-Ray diffraction
XPS: X-ray photoelectron spectroscopy
TEM: Transmission electron microscopy
S: Sulfur
Mo: Molybdenum
H_3_O^+^: Hydronium ion
b: Tafel slope
F: Faraday constant
R: Ideal gas constant
α: Symmetry factor
T: Temperature
CV: Cyclic voltammetry
N_2_: Nitrogen
PBS: Phosphate-buffered saline
KOH: Potassium hydroxide
*R*_1_: Solution resistance
*R*_2_: Charge transfer resistance
Q: Constant phase element
C: Double-layer capacitance
*R*_3_: Resistance
CB: Conduction band
VB: Valence band
$$\tau_{r}$$: Electron lifetime
*f*_*p*_: Frequency
